# Large-scale changes in cortical dynamics triggered by repetitive somatosensory electrical stimulation

**DOI:** 10.1186/s12984-019-0520-1

**Published:** 2019-05-24

**Authors:** April K. Hishinuma, Tanuj Gulati, Mark J. Burish, Karunesh Ganguly

**Affiliations:** 1 Neurology & Rehabilitation Service, San Francisco Veterans Affairs Medical Center, San Francisco, CA USA; 2 Department of Neurology, University of California, San Francisco, San Francisco, CA USA; 3 Department of Neurosurgery, The University of Texas Health Science Center at Houston, Houston, TX USA; 4 Department of Biomedical Sciences and Neurology, Cedars-Sinai, Los Angeles, CA USA

**Keywords:** Somatosensory electrical stimulation (SES), Peripheral nerve, Spiking dynamics, Motor cortex, Low frequency oscillations

## Abstract

**Background:**

Repetitive somatosensory electrical stimulation (SES) of forelimb peripheral nerves is a promising therapy; studies have shown that SES can improve motor function in stroke subjects with chronic deficits. However, little is known about how SES can directly modulate neural dynamics. Past studies using SES have primarily used noninvasive methods in human subjects. Here we used electrophysiological recordings from the rodent primary motor cortex (M1) to assess how SES affects neural dynamics at the level of single neurons as well as at the level of mesoscale dynamics.

**Methods:**

We performed acute extracellular recordings in 7 intact adult Long Evans rats under ketamine-xylazine anesthesia while they received transcutaneous SES. We recorded single unit spiking and local field potentials (LFP) in the M1 contralateral to the stimulated arm. We then compared neural firing rate, spike-field coherence (SFC), and power spectral density (PSD) before and after stimulation.

**Results:**

Following SES, the firing rate of a majority of neurons changed significantly from their respective baseline values. There was, however, a diversity of responses; some neurons increased while others decreased their firing rates. Interestingly, SFC, a measure of how a neuron’s firing is coupled to mesoscale oscillatory dynamics, increased specifically in the *δ*-band, also known as the low frequency band (0.3- 4 Hz). This increase appeared to be driven by a change in the phase-locking of broad-spiking, putative pyramidal neurons. These changes in the low frequency range occurred without a significant change in the overall PSD.

**Conclusions:**

Repetitive SES significantly and persistently altered the local cortical dynamics of M1 neurons, changing both firing rates as well as the SFC magnitude in the *δ*-band. Thus, SES altered the neural firing and coupling to ongoing mesoscale dynamics. Our study provides evidence that SES can directly modulate cortical dynamics.

## Background

Somatosensory input is essential for skilled movements [1–3]; this is particularly true for dexterous movements [1, 4–6]. Interestingly, the somatosensory system has been shown to experience relatively rapid bidirectional changes in organization as a result of repetitive manipulations of peripheral inputs. Consistent with this notion are seminal studies in both animals and humans which demonstrated that reductions in sensory feedback, either by denervation or ischemic nerve block, induced changes in motor representations [7, 8].

Studies have also shown that increases in afferent input by stimulating peripheral pathways (i.e. repetitive somatosensory electrical stimulation or SES) can alter sensorimotor representations of the stimulated body part [9, 10]. One of the first studies examining this neuromodulation method found that sensory stimulation of oral structures resulted in prolonged changes in excitability as well as an increase in the area of representation determined using functional imaging [11]. Consistent with these results are studies demonstrating that altered patterns of physical contacts to the fingers can also persistently reorganize sensory maps [12, 13]. Importantly, repetitive SES has also proven to be a promising therapeutic tool for motor rehabilitation [10, 14–16].

In both humans and rodents, SES can increase excitability as measured by responses to transcranial magnetic stimulation (TMS) pulses [9, 17]. Past studies have used non-invasive measures to examine cortical excitability such as motor evoked potentials (MEPs) with TMS [9, 17] and cortical reorganization using blood oxygenation signals [11]. It remains unclear what are the precise mechanisms underlying these changes. For example, the observed change in the evoked MEPs following SES may occur without changes in brainstem electrical stimulation-evoked potentials or spinal reflexes [9, 18, 19]. This suggests the possibility that the cortex may be an important site of plasticity. While our recent study showed that SES can also modify low-frequency dynamics as measured using electroencephalogram (EEG) [20], it remains unclear if these changes are local to cortex. Invasive electrophysiology offers one method to assess if SES can directly alter local motor cortical dynamics.

While the body of literature summarized above has provided important mechanistic insight, little is known about how SES interacts with ongoing cortical dynamics at the level of single neurons and groups of neurons, or neural ensembles. Single neurons are a fundamental unit of the nervous system. The coordinated firing of neural ensembles, e.g. co-firing of neurons in a temporally coupled manner, is now also recognized as an important module for information processing [21–26]. In addition, oscillations may provide a mechanism for dynamic coordination of ensembles across motor and sensory areas [21–25, 27]. Oscillations likely reflect synchronized rhythmic excitability linked to coordinated firing of neurons [28]. Our collective understanding of both single neuron and ensemble firing patterns has greatly improved our understanding of how neural activity patterns underlie complex sensory and motor behaviors. Similarly, it is likely that such activity may play an important role in driving neural plasticity after injury and during neuromodulation using methods such as SES.

The goal of this study was to develop a model of the cortical effects of SES using high-resolution, invasive recording of neurons. We were particularly interested in understanding the diversity of single neuron responses to SES. It is unlikely that all neurons respond identically to a given perturbation. This may be, in part, the result of the multiple cell-types in a given region and the diversity of network connectivity for single neurons [29]. We also wanted to compare changes in neural activity related to larger scale network oscillatory activity. More specifically, we examined the effects of SES on primary motor cortex (M1) at the level of single neuron firing rates as well as the neural coupling to ongoing spontaneous oscillations. We found that SES could independently change both the firing rate and the phase locking, i.e. the consistency of the neural firing relative to oscillatory dynamics. Together, our results provide evidence that SES can directly modulate neural dynamics in M1.

## Methods

### Animal and surgery preparation

All animal procedures were in accordance with protocols approved by the Institutional Animal Care and Use Committee at the San Francisco Veterans Affairs Medical Center. Adult male Long Evans rats (*n* = 8, 250-400 g, ~ 8 weeks old, Charles River Laboratories) were housed in a 12 h light:12 h dark cycle with lights out at 6:00 AM and were kept under controlled temperature. One animal was excluded from the study due to significant recording drift and electrical noise in the recording, thus *n* = 7 animals were used for the analysis shown. Animals were initially anesthetized using a ketamine/xylazine cocktail (85 mg/kg ketamine, and 10 mg/kg xylazine), with supplemental ketamine (at half of the induction dose) given every 40–60 min as needed to maintain a stable anesthetic level, and also to maintain anesthesia at stage III characterized by predominantly slow oscillations. Moreover, 0.05 mg/kg of atropine was given separately to counter respiratory and cardiac depression, and decrease secretion. Animals were sacrificed at the end of the recordings.

### Somatosensory electrical stimulation and electrophysiology

**Fig. 1:**
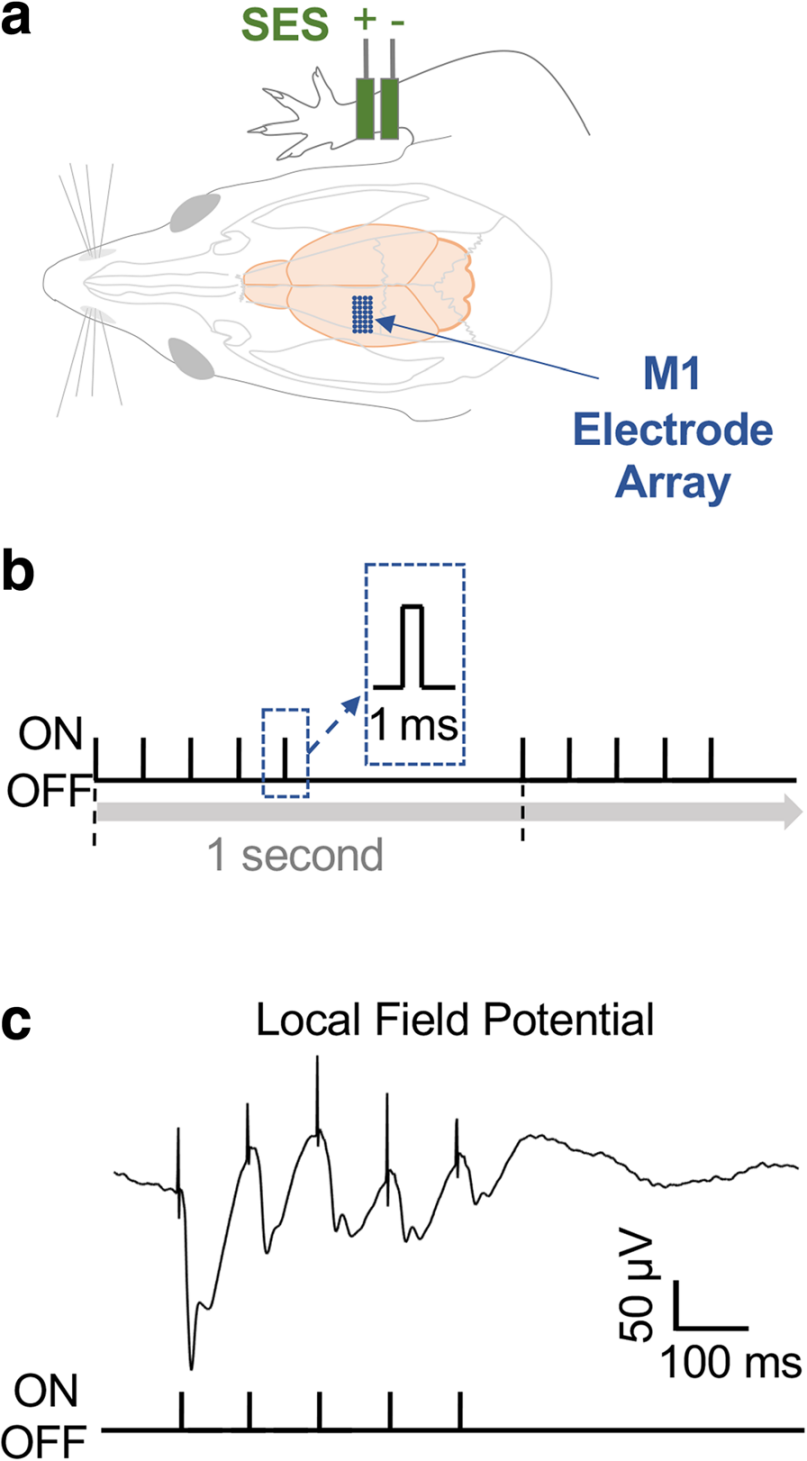
Schematic of the Experiment**. a**, Somatosensory electrical stimulation was applied directly to the distal forelimb while neural activity was recorded under anesthesia. **b**, Schematic of the stimulation paradigm. **c**, Averaged evoked potential in the local field potential during SES

After anesthesia induction, transcutaneous stimulation electrodes were clipped near forelimb peripheral nerves (medial, ulnar, and radial nerve), in the configuration noted in Fig. 1a. These copper metal clips were wrapped around the forelimb and then connected to a Multi-Channel Systems Stimulus Generator (MCS STG4000 series) to deliver transcutaneous stimulation. SES current parameters were set by determining the maximum amount of current where no evoked movement in the forelimb was seen (typically 300–750 μA currents).

Following a craniotomy and a durectomy procedure, either 64-channel custom probes in a tetrode configuration (*n* = 5, 1 X 4/8, Neuronexus, MI) or 32 channel tungsten microwire arrays (*n* = 2, MEAs, Tucker-Davis Technologies or TDT, FL) were implanted using precise stereotactic measurements into layer 5 of motor cortex (1200–1500 μm deep; + 1.5 to + 2.0 anterior to bregma and + 2 to + 3.5 lateral from midline) to record extracellular neural activity. In general, tetrodes allow better isolation of single neurons. However, as our microwire recordings also demonstrated identical findings, we have grouped the results together.

Spike data was sampled at 24414 Hz and LFP data at 1018 Hz. ZIF–clip based analog headstages with a unity gain and high impedance (~ 1 MΩ) were used. Unsorted multi-unit, single-unit, and LFP data were then recorded from 30 min to 1 h to ensure stability of recordings and to minimize drift during stimulation experiments. Then a baseline period of neural activity (~ 30–60 min) was recorded, followed by a recording of neural activity during SES. The stimulation paradigm was 5 single pulses (square pulse width, 1 ms) at 10 Hz over 500 ms, i.e. with a 1% duty cycle. This was immediately followed by 500 ms of no stimulation. This pattern of 10 Hz stimulation and no stimulation was repeated on a 1 Hz pattern (30 min for *n* = 4, or 60 min for *n* = 3 animals, current magnitude: 564.29 ± 57.46 μA, Fig. 1b). After SES stimulation was finished, post recording of neural activity was used to assess the effects of stimulation lasting ~ 30–60 min.

### Data analysis

#### LFP and single-unit analyses

Analyses were conducted using a combination of custom-written routines in Matlab 2015a/2017b (MathWorks, Natick, MA), along with functions and routines from the Chronux toolbox (http://chronux.org/). Pre-processing steps for LFP involved: removing periods of artifacts (removing broken channels, and noisy segments of LFPs based on offline visual inspection); taking the median signal (at every time point the median signal across electrodes was calculated); and z-scoring this signal (i.e. removal of the mean value, μ, of the signal, X, and dividing by the standard deviation, σ, z-scored LFP = [X–μ]/σ). Median referencing was used to remove any volume conducted signals and to thereby focus on signals local to M1.

Single units were sorted using Plexon Offline Sorter (Plexon, Dallas, TX). Single units and LFPs were used to calculate spike-field coherence (SFC) using chronux functions. SFC measures phase synchronization between the LFP and spike times as a function of frequency; its magnitude is a function of frequency and has a value between 0 and 1 [22]. For its calculation, the pre- and post-stimulation time segments were first time matched to the shortest recording period, then segmented into 10 s segments, and then the coherency measured was averaged across segments. The average time series used for analysis was 46.8157 ± 6.5765 min. For the multitaper analysis, we used a time-bandwidth (TW) product of 10 with 19 tapers. To compare coherences across groups, a *z-*score was calculated using the programs available in the Chronux Toolkit. Coherence between activity in two regions was calculated and defined as

$$ {C}_{xy}=\frac{\mid {R}_{xy}\mid }{\sqrt{\mid {R}_{xx}\mid}\sqrt{\mid {R}_{yy\mid }}} $$

where *R*_*xx*_ and *R*_*yy*_ are the power spectra and *R*_*xy*_ is the cross-spectrum. Spectral analysis was calculated in segmented time periods pre- and post-stimulation and averaged across these epochs. Mean coherence was calculated across the *δ*-band (0.3–4 Hz, i.e. all values in the range were averaged together), θ-band (6–10 Hz), α-band (8–15 Hz), β-band (18–25 Hz), γ-band (30–60 Hz). For the frequency band analysis, statistical analysis was performed on the average coherence estimates of each frequency band’s respective pre-SFC and post-SFC values (see section below). We also equaled the number of spikes in the pre- and post-stimulation period to account for the changes in firing rates [30]. The power spectrum of the LFP channels used in the coherence calculation, as well as for overall LFP power change in pre- and post-stimulation, was also determined using the multitaper method. For spiking analyses, sorted spikes were binned at 50 ms. A significant change in firing was estimated by calculating the mean post-stimulation firing rate and checking if it was outside of the 95% distribution of pre-stimulation firing rate distribution. Some analyses were further filtered down by choosing high signal-to-noise ratio (SNR) units. To clearly identify units with stable waveforms and high amplitudes, we measured SNR using the following equation:

$$ SNR=\frac{A}{2\ast {SD}_{noise}} $$

Where *A* is the peak-to-peak voltage of the averaged spike waveform and *SDnoise* is the standard deviation of the “noise”, or the baseline fluctuations in the voltage during the first 245 microseconds of the saved waveform snippet [31].

#### Spike width analysis

We grouped neurons based on the width of the recorded spikes. Spike width was calculated by finding the distance between the peak of the waveform and its valley. Past studies have demonstrated that spike width can distinguish putative fast spiking interneurons and pyramidal neurons [27, 31]. To specify a cutoff, we applied k-means to the entire neuronal population. In general, our results were concordant with this previous literature. We thus used values of 100–400 μs for narrow-width, putative interneurons and 500–1000 μs for broad-width, putative pyramidal neurons.

### Statistical analysis

Parametric statistics were used in this study, and each test was implemented within MATLAB. We used *t*-tests for comparison of power between pre- and post- SES sessions, as well as *t*-tests for the comparison of SFC pre and post-SES averaged across each common frequency band used in previous literature (*δ*-band, θ-band, α-band, β-band, γ-band) [31]; we used a Bonferroni correction for multiple comparisons. We used Pearson’s correlation and linear regression to evaluate trends between changes in firing rate and SFC after SES. The linear mixed-effects model (implemented using MATLAB fitlme) was used to compare the differences in SFC and firing rate in all units in Fig. 3f/g, and for the broad and narrow-width neurons in Fig. 4b. This model accounts for the fact that units, channels, or trials from the same animal are more correlated than those from different animals and is more stringent than computing statistical significance over all units, channels, and trials.

## Results

Long Evans rats (*n* = 7) were implanted with either microwire (*n* = 2) or tetrode (*n* = 5) arrays in M1 (Fig. 1a). Stimulation was then applied to the distal forearm peripheral nerves (30 min for *n* = 4 animals, 60 min for *n* = 3 animals, current magnitude: 564.29 ± 57.46 μA). We found that the motor evoked response was clearly visible in the LFP and showed a large deflection during the train of pulses at 10 Hz that lasted 500 ms, i.e. with a 1% duty cycle (Fig. 1c). As expected, there was a decrement in the response within each train [32].

### Firing rate changes

**Fig. 2:**
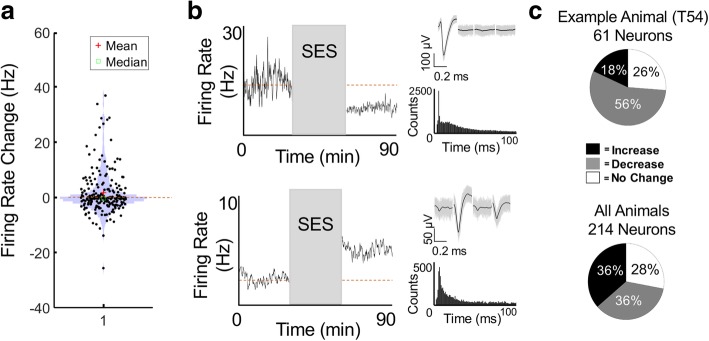
Changes in Firing Rate after SES. **a**, Violin plot of the firing rate changes for all neurons. The red cross represents the mean (1.7918); green triangle is median (− 0.2338). **b**, Example of either a significant decrease (*p* < 0.05; top) or increase (*p* < 0.05; bottom) in firing rate after SES. Also shown are tetrode waveforms and the interspike intervals. The dotted lines represent the mean during the pre-stimulation period. **c**, Percentage of neurons which significantly increased, decreased, or had no change for one animal (top) and for all animals (*n* = 7; bottom)

We first examined if SES altered the firing rate of neurons in M1 (Fig. 2) and compared changes in firing rate relative to a pre-stimulation baseline period. The overall population was widely distributed and the mean change (1.791 Hz) and median change (− 0.2338 Hz) were close to a baseline value of 0. Examples of both a significant increase (mean pre = 2.603 Hz, mean post = 5.472 Hz, *p* < 0.05) and a decrease (mean pre = 14.198 Hz, mean post = 7.603 Hz, *p* < 0.05) in firing rate are shown. In general, all animals exhibited a firing rate change in the majority of the recorded neurons after SES (i.e. > greater than 50% with a net change in firing rate at 30 min post stimulation). In an example animal T54, 56% of its units decreased their firing rate, while 18% increased their firing rates (Fig. 2b). At a population level (*n* = 214 neurons), we found that while 36% of neurons exhibited an increase in firing (mean pre = 5.93 Hz, mean post = 14.93 Hz), 36% experienced a reduction in firing rate (mean pre = 8.63 Hz, mean post = 4.64 Hz), and 28% showed no change (mean pre = 6.77 Hz, mean post = 6.52 Hz) (Fig. 2c). Regardless of the length of the time period recorded and analyzed (30–60 min), we saw a significant change relative to the baseline across all animals in neurons that either significantly increased (*p* < 10^− 04^) or decreased (*p* < 10^− 19^) their firing rates. Together, these results indicate that SES can have persistent, but diverse effects on single neuron firing rates within M1.

### Spike-field coherence changes

**Fig. 3:**
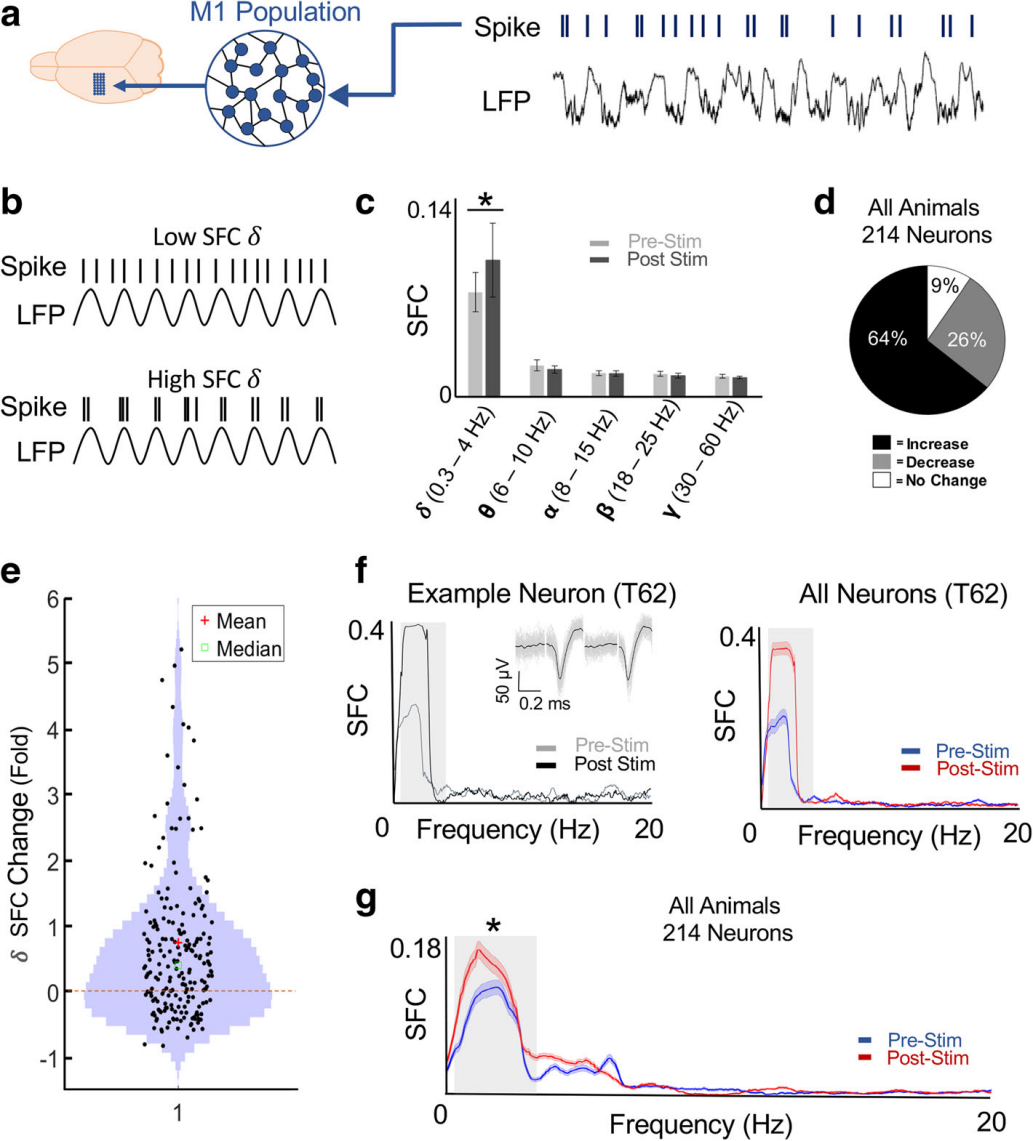
Changes in Spike Field Coherence (SFC) after SES. **a**, Schematic depicting neural spikes relative to LFP recordings from M1. **b**, Schematic of the relation of spiking to LFP for variations in the SFC. **c**, Comparison of the averaged SFC across each frequency band (see Methods) for all units before and after SES. (*p < 0.001). Error bars represent the standard error of the mean or SEM. **d**, Percentage of neurons which significantly increased, decreased, or had no change for all animals (*n* = 7). **e**, Violin plot of the SFC fold change relative to baseline for all neurons. A value of 1 represents a doubling of the SFC. **f**, Example single neuron and all neuron SFC plot for one animal. The grey box highlights 0.3–4 Hz band. Error bars are SEM. **g**, Mean SFC plot for all animal including all neurons (*n* = 214, *p < 0.001). Follows convention from **f**

We also investigated whether SES persistently modulated the synchronization between LFP and spike times as a function of frequency, i.e. spike-field coherence or SFC (Fig. 3) [25, 33]. We recorded both single unit spiking and LFP from the population of M1 units (Fig. 3a). SFC is a measure of how consistently a given unit fires relative to the phase of the median LFP (Fig. 3b). The only frequency band that showed a significant change after SES was the *δ*-band (Fig. 3c, mean change for 0.3–4 Hz *δ*-band pre- vs post-stimulation, t-test with Bonferroni correction, *p* < 10^− 09^). The θ-band (6–10 Hz), α-band (8–15 Hz), β-band (18–25 Hz), and γ-band (30–60 Hz) did not show any significant changes (*p* > 0.05).

At a single neuron level, 64% of the units increased, 26.4% decreased, and 9.6% had no change in the *δ*-band SFC (Fig. 3d). At a population level, the majority of neurons demonstrated an increase in the *δ*-band SFC relative to the baseline period (Fig. 3e). Figure 3f shows a representative change in the SFC in the low frequency, *δ*-band (0.3–4 Hz) of a single neuron; this was also evident on average for all neurons recorded in that animal. When also examining all units (*n* = 214) from all seven animals, we again found evidence for a significant SFC increase in the lower frequency band (mixed-effects model which takes into account that multiple neurons were recorded from the same animal, Fig. 3g, *p* < 10^− 05^) [34]. This indicates that after SES, neural firing was significantly more likely to be phase-locked to low-frequency oscillatory dynamics.

#### Narrow and broad spiking neurons

**Fig. 4:**
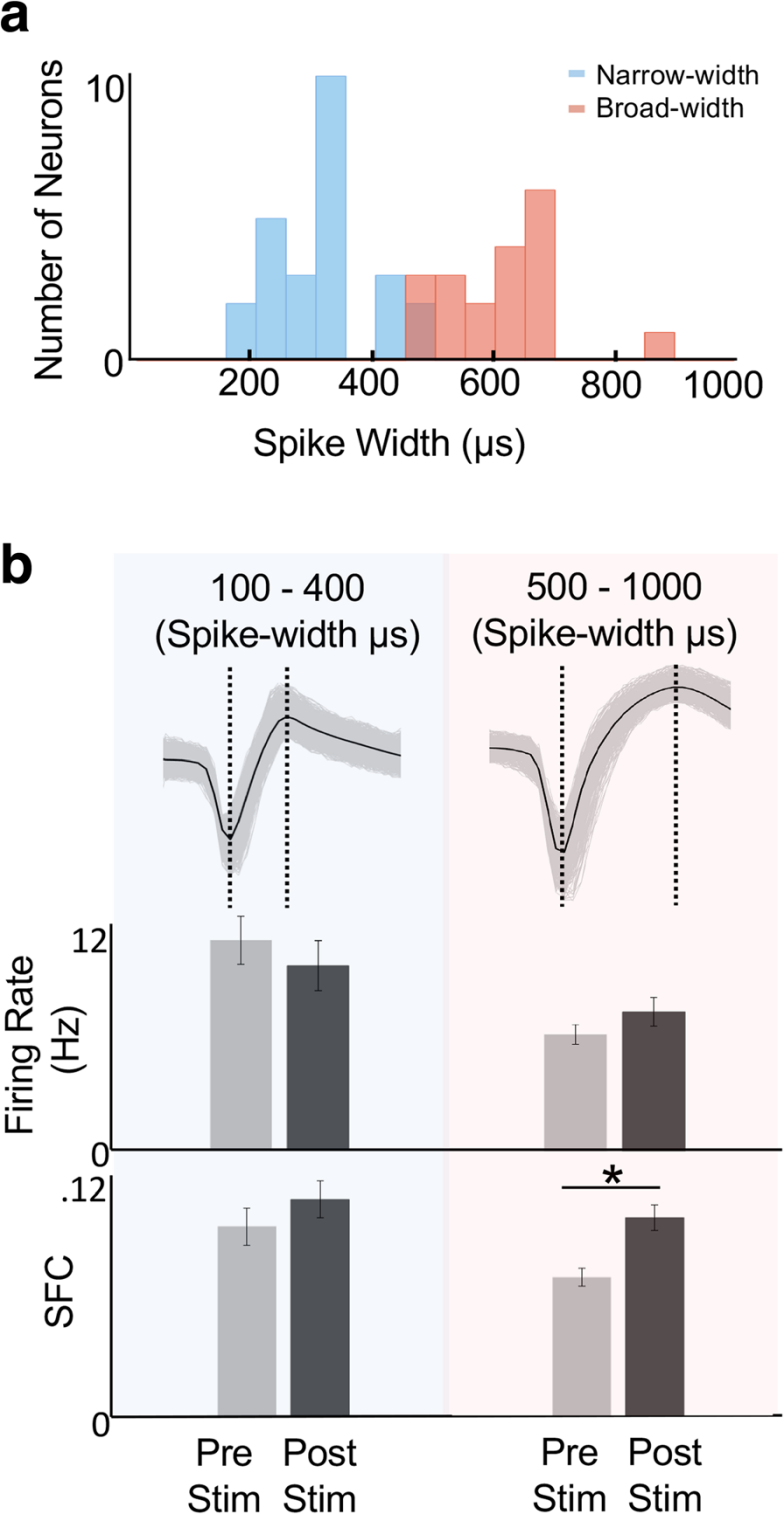
Comparison of Broad and Narrow-Width Spiking Units. **a**, Example animal’s distribution of neurons classified by spike widths (*n* = 46). The color coding is based on k-means clustering. **b**, Differences in the spiking activity and SFC for narrow-width (left blue column) and broad-width (right red column) (*p < 0.001)

We further investigated the differences in firing rate and SFC by classifying neurons into two distinct groups: narrow-spiking, putative interneurons (100–400 μs), and the broad-spiking, putative pyramidal neurons (500–1000 μs) [27, 31]. Figure 4a shows an example animal’s distribution of neuron spike widths; the color labels are based on a k-means classification. Interestingly, broad- spiking neurons demonstrated a robust increase in the SFC after SES (mixed linear model, *p* < 10^− 06^); there was no change in firing based on this classification. In contrast, narrow-spiking neurons did not show significant changes in either firing rate or SFC after SES. This implies that putative pyramidal neurons might be a main driver of the increase in SFC in the *δ*-band after SES.

### Power spectral density

**Fig. 5:**
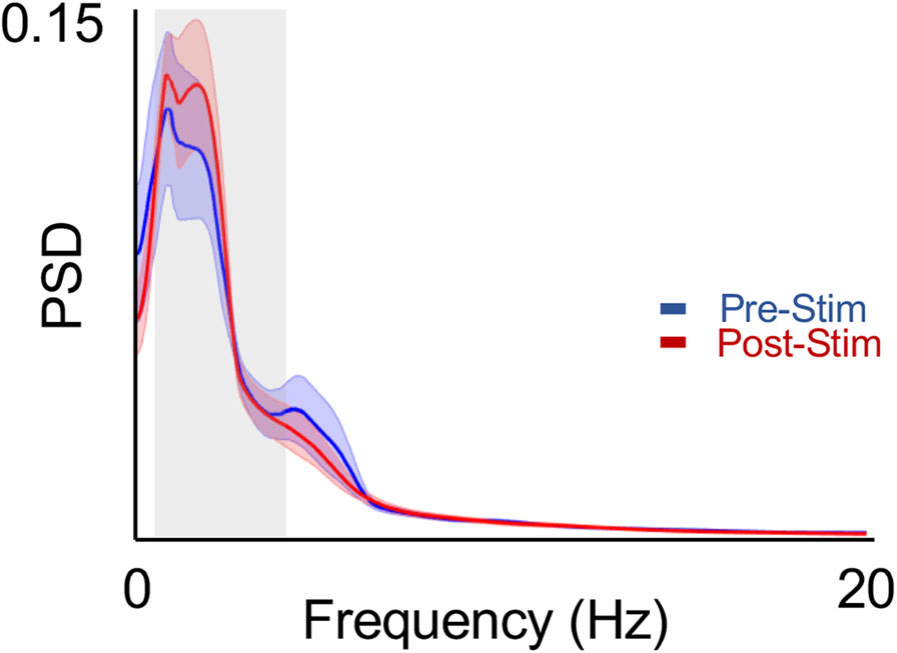
LFP Power Before and After SES. Shows the power spectrum of the LFP prior to and after SES. There was no significant relationship observed

We also examined if global changes to the LFP were also evident. The LFP is widely believed to represent an aggregate mesoscale measurement of activity [21]. There was not a significant change in the LFP power (Fig. 5).

### Firing rate and SFC changes are independent

**Fig. 6:**
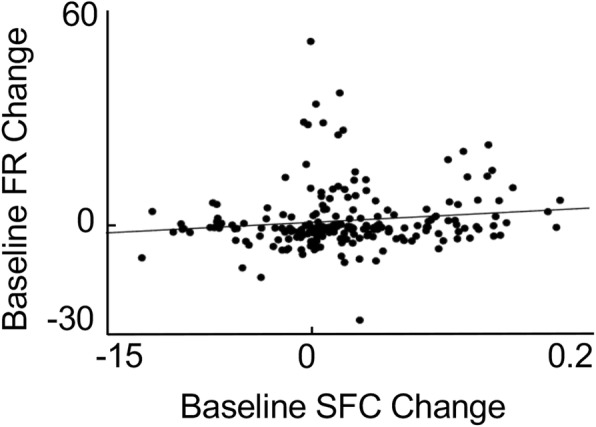
Comparison of Changes in Firing Rate versus SFC. Plot shows correlation of single neuron changes in firing rate versus the corresponding SFC change. There was not a significant relationship between the two (r = 0.13, *p* > 0.05). Line was generated using linear regression

As shown above, SES significantly modulated both the firing rates and the *δ*-band SFC. While we used methods to account for changes in firing rates (see Methods), it is possible that the SFC changes were co-regulated with the change in firing rate. We thus examined the relationship between the two variables. Interestingly, the firing rate and *δ*-band SFC were not significantly correlated with one another (Fig. 6, r = 0.1300, *p* > 0.05). This suggested that the effects of SES on the firing rate and the SFC were independent of each other.

## Discussion

We found that SES can induce persistent M1 plasticity lasting at least 30–60 min after the end of stimulation; over half of the neural population significantly changed its firing rate in response to SES. Moreover, phase locking of firing to mesoscale oscillatory dynamics was significantly modulated in a manner that was independent of the direction of change in firing rate. The most prominent SFC increase occurred in the low frequency range; there was not a concomitant change in LFP power. Together, these finding suggests that SES can directly modulate M1 dynamics.

### Relation to previous models of SES

Studies have previously shown that SES can apparently alter both the sensorimotor representations of the stimulated body part as well as excitability [9, 10, 17]. Changes in sensorimotor representations have been primarily examined using functional imaging [11], which is an indirect measure of neural activity. Moreover, in both humans and rodents, SES has also been shown to increase excitability as measured by responses to TMS pulses [9, 17]. The main uncertainty was whether M1 is directly affected by SES.

Our results add to this body of literature by demonstrating three main points. First, SES can directly modulate the activity patterns of M1; this is demonstrated by the changes in firing rates of single neurons. Second, our findings of a diversity of neural firing changes suggest a more complex neural response to SES. A better understanding of the diversity of responses and their underlying neural basis (e.g. neural connectivity, cell-types) might help improve the efficacy of SES. Third, our results suggest two possible mechanisms of SES. Namely, there was a change in spontaneous firing rate as well as coupling to mesoscale dynamics.

### Somatosensory electrical stimulation and neural plasticity

SES induced plasticity appears to be experienced differentially by the large sets of M1 neurons recorded; while a majority of the neurons experienced a change in firing rate, the extent and the direction of change was variable. Moreover, the changes in firing rate appears to equally affect both putative interneurons and pyramidal neurons. What are the potential mechanisms that can account for the diversity of changes in neural firing? On a macroscopic level, SES evoked deflections in the M1 LFP during stimulation (Fig. 1c). This is consistent with past work showing that sensory inputs can directly influence motor areas [35–37]. The reduction in response with each pulse is also consistent with the adaptation evident during sensory stimulation [32]. It is quite likely that the observed input also triggered synchronous spiking in M1. Thus, it is possible that the extent that a single neuron participated in the synchronous spiking during SES could account for the observed direction of change. It is possible that repetitive stimulation of sensory inputs to an area can result in short-term homeostatic regulation of network dynamics [38–40].

SES could also trigger activity-dependent synaptic plasticity [41, 42]. In general, brief periods of activity can trigger long-term potentiation and long-term depression that depends on the specific patterns of activation [38, 43]. Such activity can also increase or decrease the intrinsic excitability of presynaptic neurons [38, 44]. This mechanism might explain the diversity of plasticity evident at the level of single neurons. It is also worth noting that emerging computational methods to quantify functional network connectivity [23] might eventually be used to predict the specific plasticity effects at a single neuron level.

Another possibility is that the observed changes in M1 firing are the result of network plasticity in the sensorimotor system. Electrical stimulation of peripheral nerves causes synchronous activation of muscle spindles and cutaneous afferents that appear to target area specific activation and reorganization in primary somatosensory areas [14, 45–47]. Moreover, SES can trigger changes in TMS-evoked MEPs [9, 17, 18]. While past work has suggested that mechanisms of plasticity below the brainstem may not account for excitability changes [9, 18, 19], it is reasonable to suppose that larger scale network dynamics are modulated [20]. In this scenario, the observed changes in M1 could be the result of plasticity at other cortical sites. For example, given the known strong connections between sensory and motor areas [3], changes at a primary sensory area could result in spontaneous firing changes at a connected site.

### Spike coupling to low frequency oscillations

The greatest change in the coupling of neural spiking to oscillatory LFP dynamics was in the *δ*-band, also known as low frequency oscillations (LFO) [22, 48]. Our results further suggest that the change in coupling or phase-locking to mesoscale dynamics is independent from the changes in firing rate. For example, at a single neuron level, changes in firing rate did not predict changes in SFC. Moreover, we observed a change in SFC for putative pyramidal neurons without a concomitant change in firing rate. It is unclear what might drive this change. The lack of a change in LFP power in the LFO range suggests that changes in input to M1 are not a main driver; LFP is widely believed to be a measure of synaptic inputs [21, 28, 29]. Changes in intrinsic excitability is certainly a possible mechanism through which neurons can be more coupled to population dynamics [38]. This might also explain the previously observed changes in M1 evoked potentials after SES [9, 17]. Alternatively, changes in local synaptic connectivity [29], i.e. as distinct from synchronous inputs to M1, could be a driver of the changes in neural coupling to population dynamics.

What might be the broader physiological consequences of SES induced changes in LFO dynamics? In general, ketamine anesthesia is known to result in such low-frequency oscillatory activity [22, 48]. However, in rodents, non-human primates and humans, LFOs have been observed at the level of spiking and LFP in the motor cortex during reaching tasks [22, 24, 48, 49]. It has been postulated that LFOs represent an intrinsic property of motor circuits that are involved in the production of fast and accurate movements. Stroke disrupts these movement related potentials in humans, which are highly correlated with motor impairments [22, 49]. LFOs are therefore a potential biomarker of restored circuit dynamics after stroke as it relates to fast and accurate skilled reaching [20, 22]. Interestingly, our recent study also found that parameters for modulation of LFOs in anesthesia also generalized to the awake state [22]. It is thus possible that the locking of spiking to LFOs is a general principle for the cortical effects of SES. In other words, SES might be particularly suited for modulating the neural dynamics linked to cortical slow oscillations. Future work can examine if SES also similarly modulates movement-related spiking in the healthy or perilesional cortex; this might be one mechanism through which SES improves function in stroke patients [20, 50].

## Conclusions

In summary, brief periods of SES induced long-lasting cortical plasticity in M1. We identified significant changes in firing rate and spike coupling to low frequency oscillations in the majority of recorded neurons. Further tailoring of these processes to identified cortical dynamics might further improve the efficacy of SES in those with motor disabilities after stroke or other acquired brain injuries [22, 50].

## Abbreviations

EEG: Electroencephalogram
LFO: Low frequency oscillation
LFP: Local field potential
M1: Primary motor cortex
MEP: Mean evoked potential
PSD: Power spectral density
SD*noise*: Standard deviation of noise
SEM: Standard error of the mean
SES: Somatosensory electrical stimulation
SFC: Spike field coherence
SNR: Signal-to-noise ratio
TMS: Transcranial magnetic stimulation
TW: Time-bandwidth
α-band: Alpha-band
β-band: Beta-band
γ-band: Gamma-band
θ-band: Theta-band
δ-band: Delta-band
